# Notes of the Week

**Published:** 1921-09-03

**Authors:** 


### NOTES OF THE WEEK.

### THE SPAHLINGER TREATMENT OF TUBERCULOSIS.

This subject is again receiving notice in the lay Press. We believe that a special medical journal will shortly publish full particulars. Not to anticipate these, we may say that no conclusive evidence of specific remedial activity is likely to be produced. The remedy *a priori* does not sound very promising, even leaving out of count the thousand and one unsuccessful precedents. Professor Spählinger is reported as saying that the vaccine part of the treatment is the more important one. This, as described by him up to now, is the use of antigens derived from various components of the tubercle bacillus, so that from successive production of partial immunities there may be built up a total immunity. But the very same principle has for some time been in use by its originator, Much, of Hamburg, whose "partigen" tuberculin treatment has been widely tried and studied in Germany, and indeed with not much final success. Like so many bacteriological measures, it has increased our knowledge of the disease, but therapeutically has fallen far below expectation, at least the expectation of its originator. It looks then as if Professor Spählinger has clapped on a serum, in order to attempt a combination of passive immunity with active immunity. This is on the lines of the old blunderbuss prescription, rather than of any new principle. We have no wish, needless to say, to deprecate continual effort and research, but it is necessary to moderate the effect on the public mind likely to result from loud sounding reports in the tendencious lay Press of these days.

### HOSPITAL WEEK AND PAGEANT: IDEAS FROM AMERICA.

In the September number of *Progress*, the journal of the Great Northern Central Hospital, Holloway, appears an interesting letter from an American correspondent in support of the proposed Hospital Week and Pageant in this country. The writer says: "On May 12 this year about 2,500 hospitals of the United States and Canada participated in National Hospital Day. . . . 250,000 people visited the hospitals on that day and were shown through the various departments. . . Many hospitals reported receipts of money, linen, etc., and others received applicants (*sic*) from girls whose interest in nursing was aroused in this way. May 12 was chosen . . . as the anniversary of the birth of Florence Nightingale. . . . We are sure that the hospitals of London can derive a great deal of benefit from such a day, and we hope next year to . . . make Hospital Day an international tribute to Miss Nightingale, and also an international movement for a better understanding on the part of the public of what hospitals mean." The plan originated with *Hospital Management*, whose editor, Mr. Mathew O. Foley, writes the above to *Progress*, and encloses the May issue of his paper which described the observance of the occasion. Mr. Foley has also offered to send to our contemporary "suggestions for publicity, and programmes which were sent to the hospitals of North America and which were most generally followed." These facts are welcome, for though Hospital Day in America is somewhat different in conception from a Hospital Week and Pageant in London, yet there is no doubt that ideas can be gained; and we hope that *Progress* will help to make them available.

### AMERICAN HOSPITAL PROGRESS.

The Government of the United States is turning to good account the army posts and barracks which are no longer needed. Several of these are to be converted into hospitals. As the posts have been abandoned for periods varying from ten to twenty months, they will require very considerable overhauling and adaptation before they can admit patients, but they are in healthy situations, and when fitted up will be of great value. One, Fort Walla Walla, situated on a plateau near the junction of the fruit and wheat belts, will accommodate 284 tuberculosis patients, while Fort Mackenzie, in Wyoming, sheltered by a range of mountains to the north, and Fort Roots, in Arkansas, on a bluff overlooking the river Arkansas, are to be dedicated to nervous mental cases. Each will accommodate 600. A naval station at Gulfport, Mississippi, has also been taken over by the Public Health Service, and will be used as a hospital. Besides adapting these Service institutions, the health authorities of the United States are erecting several new hospitals, or purchasing existing places for adaptation to hospital uses. One of these, the Speedway Hospital, at Chicago, will take in 1,000 patients, while farther west, at Portland, Oregon, a smaller hospital is now ready to accommodate 164. In Kentucky, a hospital to receive 500 tuberculous patients is being prepared at Dawson Springs, and at Colfax, Iowa, a hotel with 130 acres of ground is being fitted up to provide for 200 patients.

### POWER TO CONTRIBUTE TO HOSPITALS.

A scheme to aid voluntary hospitals has been prepared by the Sheffield Corporation, under which employees agree to contribute one penny per week out of each £1 of their wages for such purpose, employers adding a sum equal to one-third of the total subscribed by the employees. When the scheme came before the Finance Committee, the Town Clerk expressed the opinion that the Corporation was not entitled to contribute towards the scheme without first securing power. In these circumstances the Corporation has now decided to seek such power in its next Provisional Order.

### THE COSTERS' HOLIDAY.

It is the amiable custom of the London Street Traders always to start on their annual outing from the doors of some London hospital, so that a collection may be made on its behalf. This year the start was made from the London Hospital, where twelve motor-coaches, containing three hundred passengers, met to convey them to Brighton for the day. The students who issued from the hospital to make a collection were handsomely rewarded; and when the party arrived at Brighton another collection was made on behalf of the hospital there. In 1920 the start was made from the doors of the Metropolitan Hospital. Next year the meet will be at St. Bartholomew's. On the present occasion Mr. G. J. Cook, who is Chairman of the Executive of all the costers' organisations, read a letter from the Secretary of the London Hospital Collecting Committee, which warmly thanked the costers for their help.

### A HINT TO BOLTON.

Provincial newspapers, when dealing with hospital affairs, usually follow humbly local leaders, and very rarely suggest any ideas to them. It is, therefore, satisfactory to find the *Bolton Journal* explaining on its own initiative the chief points of the "Sussex" scheme for hospital provision, and suggesting that its adoption might solve the kindred needs of the people of Bolton and of the Bolton Infirmary. With a waiting-list of 305, and collections reduced by the coal strike, to say nothing of the need for impressing the new local committee, the time has surely come for considering either the Sussex or Oxford plan if Bolton has no local alternative.

### THE SALE OF PROPERTY.

That the market for the selling of property is still a good one is shown by the action of St. Bartholomew's Hospital. It has sold four fully licensed houses, which have hitherto brought in a rental of £455. The sale produced a sum of £21,950, which produces an income of £1,269. To replace the difference between the previous value and the capital realised a sinking fund has been formed, which will involve a payment of £200 a year for thirty years.

### THE JOHANNESBURG HOSPITAL INQUIRY.

The inquiry into the administration of the Johannesburg Hospital has resulted in the Commission (with one dissentient) registering its opinion that it is monstrous that sweeping charges should be made against hospital officials with no facts to substantiate those charges. The Commission finds the internal organisation and management of the hospital are deserving of high praise, and it pays a high tribute to the zeal, ability, courage, and honesty of the Medical Superintendent, Dr. Mackenzie, to whom and "to his able heads of departments is due a debt of gratitude, not merely of Johannesburg but of the Transvaal and the Union."

### AN M.P.'S CONVERSION.

Speaking at a demonstration on behalf of the funds of Rotherham Hospital, Yorkshire, Mr. T. W. Grundy stated that he had been speaking at hospital gatherings for thirty-seven years. It had been said that he advocated State hospitals because everyone would then contribute to their maintenance. If he had ever had a tendency in that direction, Mr. Grundy proceeded, since he had been in Parliament he had altered it considerably. From his knowledge of some State-managed departments, he much preferred the old-tried voluntary method. This is an interesting confession, and reminds us that the opinions of most people are misfits, and that practical experience of systems often advocated would completely change the advocate's attitude in regard to them. It is doubtless a counsel of perfection, but the fact remains that wise people confine their opinions, as far as possible, to matters of which they have personal knowledge, and gladly admit to having no opinion on the rest.

### THE PATIENTS' DIET AT NORTHAMPTON.

The diet and meals of the patients at Northampton General Infirmary have been examined in detail by the house committee, which has presented to the Governors its report, together with a memorandum by Mr. James Gribble, who made a complaint at the quarterly court in May. After paying three surprise visits, Mr. Gribble declared that the meals and service were "very satisfactory," except that the eyes were left in the potatoes, that only one vegetable was served, and that haddock was the fish on each of his visits. He suggested that hake, cod, or plaice would be nicer, and no more expensive. Save for the complaint of one vegetable only, the patients were satisfied. He understands that luncheon, tea, and supper have been improved; and if the present standard is maintained he does not think that many complaints will be heard in future. Mr. G. W. Beattie, who accompanied Mr. Gribble on one occasion, also recorded the complaint that "only one vegetable was ever served," but heard no others, and was himself satisfied with the diet. The medical board, having compared the patients' diet with that at other hospitals, and having exercised its power to order additions when necessary, thinks that any further variety should be left to the house committee and to the matron, Miss Amelia Smith.

### CHILD CRITICS IN FEVER HOSPITALS.

Fever hospitals, like asylums, usually receive more criticism than praise. It is, therefore, pleasant to read the appreciation of a father whose three children had lately to be removed, during the epidemic, to the Edinburgh City Hospital. His testimony is valuable for two reasons. First, he records that the compulsory removal of his children was much against his will and their own. Secondly, alas! his youngest child died a week after admission. The two others were, happily, restored to him after their recovery. He and his wife record the sympathy which they received, and his children, he says, "look back with pleasure to the few weeks spent in hospital." Probably the patients' point of view can be expressed no more sincerely and truthfully than by children. If they have no fault to find, the hospital and staff have good reason to be proud.

